# Gamma-Secretase Inhibitor Treatment Promotes VEGF-A-Driven Blood Vessel Growth and Vascular Leakage but Disrupts Neovascular Perfusion

**DOI:** 10.1371/journal.pone.0018709

**Published:** 2011-04-14

**Authors:** Mattias Kalén, Tommi Heikura, Henna Karvinen, Anja Nitzsche, Holger Weber, Norbert Esser, Seppo Ylä-Herttuala, Mats Hellström

**Affiliations:** 1 Department of Immunology, Genetics, and Pathology, Uppsala University, Uppsala, Sweden; 2 Department of Biotechnology and Molecular Medicine, A. I. Virtanen Institute for Molecular Sciences, University of Eastern Finland, Kuopio, Finland; 3 ProQinase GmbH, Freiburg, Germany; Stockholm University, Sweden

## Abstract

The Notch signaling pathway is essential for normal development due to its role in control of cell differentiation, proliferation and survival. It is also critically involved in tumorigenesis and cancer progression. A key enzyme in the activation of Notch signaling is the gamma-secretase protein complex and therefore, gamma-secretase inhibitors (GSIs)—originally developed for Alzheimer's disease—are now being evaluated in clinical trials for human malignancies. It is also clear that Notch plays an important role in angiogenesis driven by Vascular Endothelial Growth Factor A (VEGF-A)—a process instrumental for tumor growth and metastasis. The effect of GSIs on tumor vasculature has not been conclusively determined. Here we report that Compound X (CX), a GSI previously reported to potently inhibit Notch signaling *in vitro* and *in vivo*, promotes angiogenic sprouting *in vitro* and during developmental angiogenesis in mice. Furthermore, CX treatment suppresses tumor growth in a mouse model of renal carcinoma, leads to the formation of abnormal vessels and an increased tumor vascular density. Using a rabbit model of VEGF-A-driven angiogenesis in skeletal muscle, we demonstrate that CX treatment promotes abnormal blood vessel growth characterized by vessel occlusion, disrupted blood flow, and increased vascular leakage. Based on these findings, we propose a model for how GSIs and other Notch inhibitors disrupt tumor blood vessel perfusion, which might be useful for understanding this new class of anti-cancer agents.

## Introduction

In the last decade dozens of new cancer drugs of the “targeted therapy” class have been introduced. These drugs, for example trastuzumab and imatinib, are based on efforts in basic research to understand the cellular mechanisms underlying cancer development. The majority of these drugs are designed to interfere with growth promoting signaling pathways, hi-jacked by the tumor cells. More recently, drugs affecting tumor growth indirectly, through inhibition of VEGF-A-driven angiogenesis, have also been introduced in the clinic [1], [2]. Based on the success of the first wave of targeted therapies, there is now a rapid development of novel modulators of cell signaling pathways, DNA-repair, proteolysis etcetera [3], [4], [5].

For more than a century, the Notch signaling pathway has been known as a critical regulator of fundamental cell fate decisions during development in *Drosophila*. Notch mainly exerts its functions through lateral inhibition, a process by which a cell - having acquired a certain phenotype - inhibits neighboring cells to take the same path of differentiation. The result is two distinct cell types, where both stem from cells with the same developmental potential [6]. In vertebrates there are five known Notch ligands (Delta-like 1, -3 and -4, Jagged 1 and -2), and four Notch receptors (Notch1-4). As in *Drosophila*, the Notch system controls development at multiple steps in almost all vertebrate tissues. Both the ligands and receptors are cell surface bound and require cell-cell interaction to trigger signaling. Furthermore, ligand-receptor interaction leads to a series of proteolytic cleavages, where the last step is mediated by the gamma-secretase membrane protein complex, which minimally consists of the four proteins Presenilin, Nicastrin, APH-1, and PEN-2. The series of proteolytic cleavages leads to translocation of the Notch intracellular domain (NICD) into the nucleus and initiation of transcription [7]. Apart from being essential for normal development, the Notch signaling system has been implicated in human hereditary cardiovascular diseases and cancer [8], [9]. For example, the Notch gene is translocated, mutated and over-expressed leading to gain of function in a large number of malignancies including cervical, head and neck, endometrial, renal, lung, pancreatic, ovarian, breast and prostate carcinoma [10], [11], [12] and reviewed in [8].

Recently, our understanding of the role of Notch signaling in angiogenesis in general, as well as in tumor angiogenesis, has improved. During normal development, Delta-like 4 (Dll4) is expressed by endothelial tip cells at the front of the angiogenic sprout and signals via Notch1 to neighboring stalk cells trailing behind, thereby suppressing differentiation of new tip cells and sprouts. The disruption of this signal, by targeting Dll4 or Notch1 either genetically or pharmacologically, leads to increased formation of endothelial tip cells, and in turn excessive angiogenic sprouting [13], [14], [15], [16], [17], reviewed in [18]. In the tumor setting the same mechanism operates so that Dll4 or Notch1 inhibition drives extreme angiogenic sprouting, which generates non-functional vasculature, in turn leading to inhibited tumor growth [19], [20], [21], [22], [23], reviewed in [24].

Thus, there are multiple reasons for designing drugs disrupting the Notch signaling pathway and various targeting approaches are under way ranging from antibodies that selectively inhibit specific Notch ligands or receptors, to low molecular weight compounds disrupting the signaling of all four receptors [25]. The latter class of compounds is based on inhibition of the gamma-secretase protein complex, and is consequently termed gamma-secretase inhibitors (GSIs). Apart from being essential for Notch signaling, the gamma-secretase facilitates catalytic cleavage of several other membrane proteins such as amyloid polypetide (APP), erbB-4, CD44, E-cadherin etcetera [26]. The metabolic activity and nutrient supply of the tumor cell might also be affected by GSI treatment since mammalian target of rapamycin (mTOR) and the glucose transporter Glut1 have been shown to be downstream of Notch signaling [27], [28]. The first GSIs in drug development were aimed at treatment of Alzheimer's disease based on the amyloid hypothesis. However, there is now an increased interest in GSIs as a potential treatment for hematological malignancies and cancer since they have been shown to be efficacious in several tumor models [29], [30].

Despite thorough investigation of GSIs in tumor models, surprisingly little effort has been directed at analyzing its effect on tumor vessel morphology and functionality. We therefore analyzed the effect of Compound X (CX), a GSI previously reported to potently inhibit Notch signaling *in vitro* and *in vivo* [31]. Here we report that CX promotes VEGF-A-driven angiogenic sprouting *in vitro* and during developmental angiogenesis. CX also suppresses tumor growth in a renal carcinoma model, leads to the formation of abnormal vessels and an increased tumor vascular density. In a model of VEGF-A-driven angiogenesis in skeletal muscle, we then demonstrate that CX promotes abnormal blood vessel growth characterized by vessel occlusion, disrupted blood flow, and increased vascular leakage. These findings might suggest that GSIs act as anti-tumoral agents, not only via direct effects on tumor growth, survival and differentiation, but also through action on the tumor vasculature.

## Results

### Compound X promotes angiogenic sprouting both *in vitro* and during mouse development

**Figure 1:**
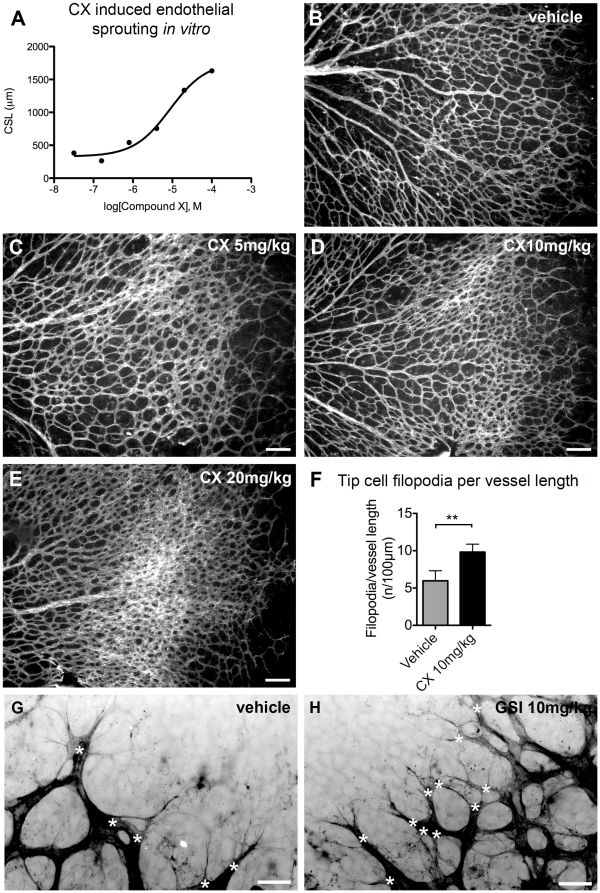
CX dose-dependently promotes angiogenic sprouting and vessel growth *in vitro* and *in vivo*. (A) Dose-response curve for CX in endothelial sprouting assay using HUVEC in type I collagen gels (EC_50_ value: 8.8 µM). Cumulative sprout length (CSL) was calculated from the ten longest sprouts from ten HUVEC spheres. (B-E) Mouse retinas analyzed at day 5 after systemic treatment of mice, between postnatal day 3-4, with vehicle, B or CX at 5 mg/kg, C, 10 mg/kg, D and 20 mg/kg, E. Endothelial cells were stained using Isolectin B4, white. There was a clear dose-dependent increase in the vascular density after CX dosing. (F) There was a 68% increase in the number of filopodia protrusions per vessel length when comparing CX-treated (10 mg/kg) retinas to vehicle (CX; 9.8 versus vehicle; 5.8 filopodia protrusions/100 µm, P-value = 0.0041). (G-H) High-magnification images of vehicle and CX-treated (10 mg/kg) retinas showing endothelial tip cells (white asterisks) with extensive filopodia protrusions, isolectin B4-positive staining in black. Error bars represent standard deviation. The scale bars in B-E are 100 µm and in G-H 25 µm. ** = P<0.01.

CX has previously been shown to potently inhibit gamma-secretase activity and Notch signaling *in vitro* and *in vivo* [31]. To test whether CX directly promotes angiogenic sprouting *in vitro*, we titrated CX in a cellular angiogenesis assay where clustered human umbilical vein endothelial cells (HUVECs) form sprouts in a three-dimensional collagen type I gel [32]. CX induced HUVEC sprouting dose-dependently with an EC_50_ of 8.8 µM. (**Figure 1**). To ascertain that GSI inhibitors affect angiogenesis similarly, we also tested four other compounds; LY450.139, Sulfonamide 565763, WPE-III-31C and DAPT, pertaining to different classes of GSI inhibitors. All compounds promoted angiogenic sprouting dose-dependently (data not shown).

We and others have previously shown that genetic or pharmacological inhibition of Notch signaling leads to increased angiogenic sprouting *in vivo* during developmental angiogenesis of the retina, reviewed in [18]. As expected, CX dose-dependently increased the vascular density of the early postnatal retina at doses between 5 and 20 mg/kg/day (**Figure 1**). Treatment with CX between postnatal day (P) three and five led to increased vascular density in the outermost two thirds of the retina, accompanied by super numerous endothelial tip cells at the sprouting vascular front in the periphery. In line with this, treatment with CX (10 mg/kg) between P 3 and P 5 led to a 68% increase in the number of endothelial filopodial protrusions at the vascular front, compared to control (**Figure 1**). Thus, the pattern of vascular formation after CX treatment was essentially morphologically indistinguishable from treatment with other GSIs such as DAPT [13]. The *in vitro* and *in vivo* doses of CX, used to potently stimulate angiogenic sprouting, were similar to the doses used to inhibit Notch signaling in prior work by Searfoss et al. [31].

### Compound X modulates the angiogenic response in a model of renal cell carcinoma

**Figure 2:**
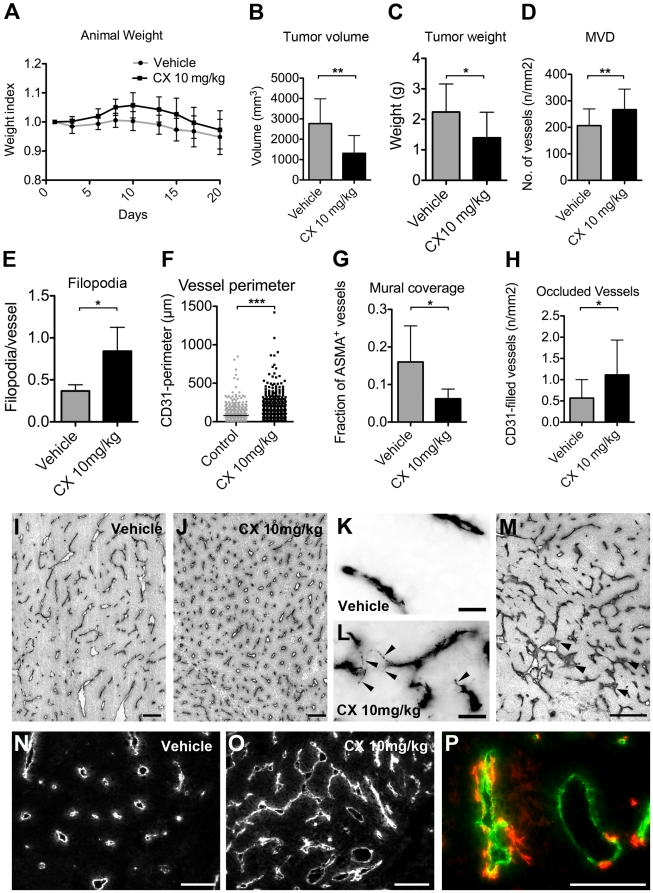
CX treatment is anti-tumorigenic and promotes angiogenic sprouting in mouse renal cell carcinoma model. At day 0 twelve mice were orthotopically inoculated with RENCA cells under the kidney capsule. (A) Mean animal weight plotted in relation to their initial weight. There was a weight reduction in the control group, 1.0 g (a loss of 5.5% of initial weight), P = 0.049 between day 1 and 20. There was no significant weight reduction in the CX- treated cohort. At day 21 the study was terminated. (B) Tumor volume was reduced in the CX- treated group by 53% of control, P = 0.0055**.** (C) Tumor wet weight was reduced in the CX- treated group by 38% of controls, P = 0.041. (D) Microvascular density (MVD) was measured as the number of CD31 positive structures per area, and was increased by 29% of control after CX treatment, P = 0.0080. (E) The number of filopodia-like protrusions from the vessels profiles were increased by 130% in CX 10 mg/kg treated tumors compared to vehicle treated tumors (CX 10 mg/kg; 0.84 versus vehicle; 0.37 endothelial protrusions per vessel profile, P = 0.017). (F) Vessel perimeter was calculated as the length surrounding the CD31^+^ blood vessel areas and there was a significant increase in the mean vessel perimeter in the CX-treated tumor as compared to control, 107 µm versus 85 µm, P<0.001. (G) The mural cell coverage was quantified as the fraction of CD31^+^ vessels with associated ASMA^+^ cells, and was 0.16 in the vehicle group and 0.06 in the CX-treated group, P = 0.048. (H) The number of occluded vessels per area was increased 1.93-fold in CX- treated tumors as compared to controls, P = 0.010. (I and J) Representative tumor fields from control and CX-treated tumors stained with CD31. Black structures are CD31 positive vessels in I-M. (K–L) A higher frequency of endothelial filopodia-like protrusions were observed after CX treatment compared to control, black arrowheads point at filopodia-like protrusions. (M) Occluded vessels were identified as large vessel structures completely filled with CD31-positive structures (arrowheads), clearly distinguished from small, and presumably, tangentially sectioned vessels. (N) Control tumors stained for CD31, white structures. (O) CD31-stained CX-treated tumors form long continuous vessels structures that seem to arise from fusion of several previously distinct vessels, e.g. center of image. (P) Example of CD31 (green) and ASMA (red) stained tumor section. Error bars represent standard deviation. Compound X: CX. Scale bars are 100 µm, except K-L which are 25 µm. Significance level were indicated * =  <0.05, ** = P<0.01. *** = P<0.001.

To assess the effect of GSI treatment on tumor growth and vessel formation in the tumor setting, we treated mice with CX (10 mg/kg/day) in a model of renal cell carcinoma. Balb/C RENCA cells were orthotopically implanted into syngenic mice at day 0 and tumors were analyzed at day 21. Prior to animal experiments we assessed the effect of CX on RENCA cell viability at escalating doses *in vitro*, and found that it had no significant effect even at the highest dose tested (10 µM), data not shown. Hence, at the doses used in the study, CX affected angiogenic sprouting but not cell viability. As seen previously in the RENCA tumor model, vehicle-treated animals lost weight over time due to their tumor burden and at day 20 the reduction was 5.5% of the initial weight. CX-treated animals did not show a significant weight reduction (**Figure 2**). At the end of the study, the primary tumor volume and wet weight were both reduced after CX treatment; 53% and 38% of control, respectively (**Figure 2**). The microvascular density (MVD) in CX-treated animals, measured as the number of CD31/PECAM1-positive structures per unit area, was increased by 29% of control. The blood vessel morphology was also altered. The vessel perimeter surrounding the CD31-positive structures in the CX-treated group was increased by 33% of control. However, the vessels were not dilated, but rather it appeared as if several blood vessels were in the process of merging into larger blood vessel structures (**Figure 2**). At the level of endothelial cells, they appeared different in CX-treated tumors compared to control. The endothelial surface was uneven and the number of filopodia-like protrusions from endothelial cells were more than doubled after CX treatment, compared to control (**Figure 2**). The mural cell coverage of the tumor vasculature was quantified as percentage of CD31-positive blood vessels that were associated with α-smooth muscle actin (ASMA) positive cells. In the vehicle treated group the ASMA-positive blood vessels were few (16%) and most of them were only partially covered. The mural cell coverage was reduced by 61% of control in the CX-treated group (**Figure 2**). Interestingly, we also observed a higher frequency of vessels filled with a CD31-positive content. In trying to differentiate such vessels from the ones having merely been tangentially sectioned, we quantified only large vessels with CD31-positive content as being “occluded vessels”, exemplified in Figure 2. Occluded vessels were seen in proximity to areas undergoing necrosis and were 2-fold more common in the CX-treated tumors compared to vehicle control (**Figure 2**). Moreover, the intraluminal occlusions were positive for the endothelial markers CD31 and podocalyxin, as well as the nuclear stain DAPI, suggesting that they are viable endothelial cells and not membranous or matrix material (**Figure S1**).

Albeit we observed significant changes in microvascular density and tumor size between vehicle and CX-treated animals, we failed to quantify any differences in proliferation (BrdU-incorporation), apoptosis (TUNEL and cleaved Caspase 3-staining), or area of necrosis, data not shown.

### Compound X disrupts VEGF-A induced neovascular perfusion

**Figure 3:**
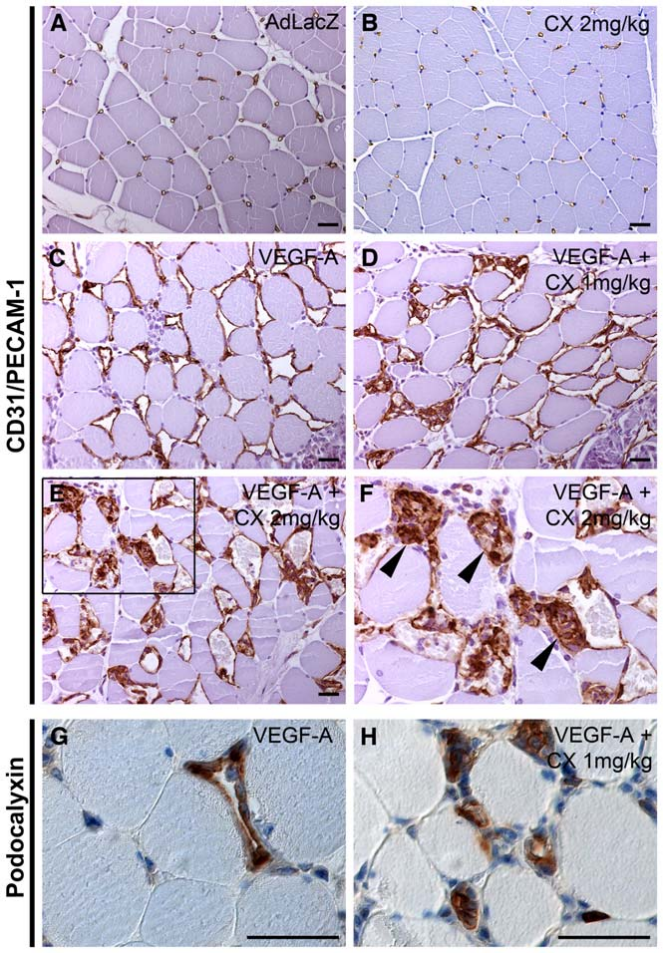
CX treatment leads to formation of abnormal angiogenesis and vessel occlusion in model for VEGF-A driven angiogenesis. (A–H) Cross section of rabbit skeletal muscle semimembranous muscle, hematoxylin staining in blue and endothelial CD31/PECAM1 or Podocalyxin staining in brown, as indicated. (A) Thin-walled capillaries are evenly interspersed in the AdLacZ-treated (control) skeletal muscle. (B) 2 mg/kg CX treatment did not alter the vascular morphology of the skeletal muscle capillaries. (C) Administration of VEGF-A_165_-isoform by adenoviral vector promotes an angiogenic response resulting in dilation and proliferation of the existing vessels, day six after gene transfer. (D–E) Addition of 1 or 2 mg/kg CX (D and E, respectively) dose-dependently abrogates the normal VEGF-A response and leads to the formation of PECAM1-positive structures occluding the vessel lumen (arrowheads, F is a magnification of E). (G–H) The endothelial marker, Podocalyxin, labeled the endothelial cells specifically in AdVEGF-A and CX-treated treated skeletal muscles, brown. (H) Podocalyxin revealed the same cellular structures occluding the vascular lumens after CX treatment as the CD31 staining. Scale bars are 25 µm.

**Figure 4:**
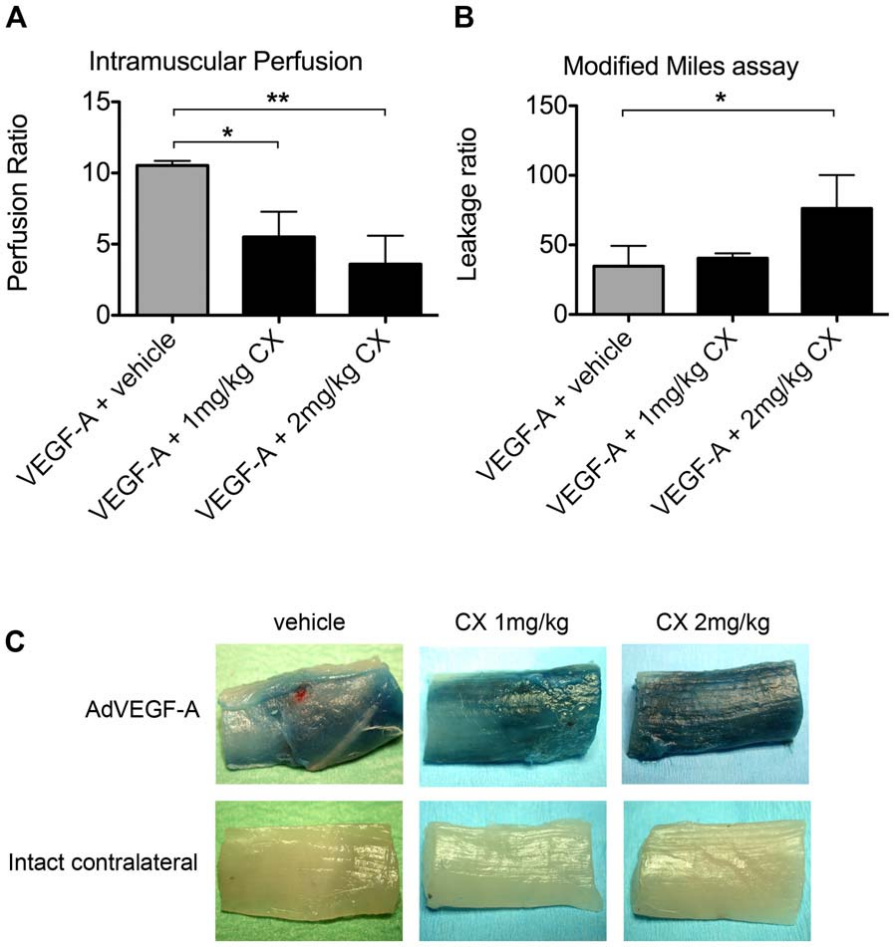
CX treatment leads to decreased perfusion and increased leakage after VEGF-A induced angiogenesis. Contrast pulse sequence (CPS) ultrasound was used to measure vascular perfusion in the semimembranous muscle. The CPS-ratio between the VEGF-A adenovirus gene transferred leg and the non-treated leg was compared. (A) VEGF-A induced a 10.3-fold increase in perfusion. There was a significant dose-dependent reduction in the CPS ratio between VEGF-A transferred leg and the control leg when CX was systemically administered. The CPS ratio was 5.5 and 3.6 after administration of 1 and 2 mg/kg CX, respectively, ANOVA P = 0.0040. (B) Evans blue was injected into the blood stream prior to extraction of the muscle tissue. The signal between the VEGF-A treated muscle versus the non-treated leg was compared. There was a 35-fold increase in Evans blue leakage after VEGF-A gene transfer, which was further increased by administration of CX. Doses of CX at 1 mg/kg or 2 mg/kg resulted in a 40- or 76-fold increase in Evans blue leakage, respectively, ANOVA P = 0.041. (C) Biopsies from the semimembranous muscle from either AdVEGF-A_165_ treated or intact leg with the systemic treatment as indicated in the figure. The blue dye comes from extravasated Evans blue that is trapped in the tissue. Compound X: CX. Error bars represent standard deviation. Significance level were indicated * = P<0.05, ** = P<0.01. *** = P<0.001.

**Figure 5:**
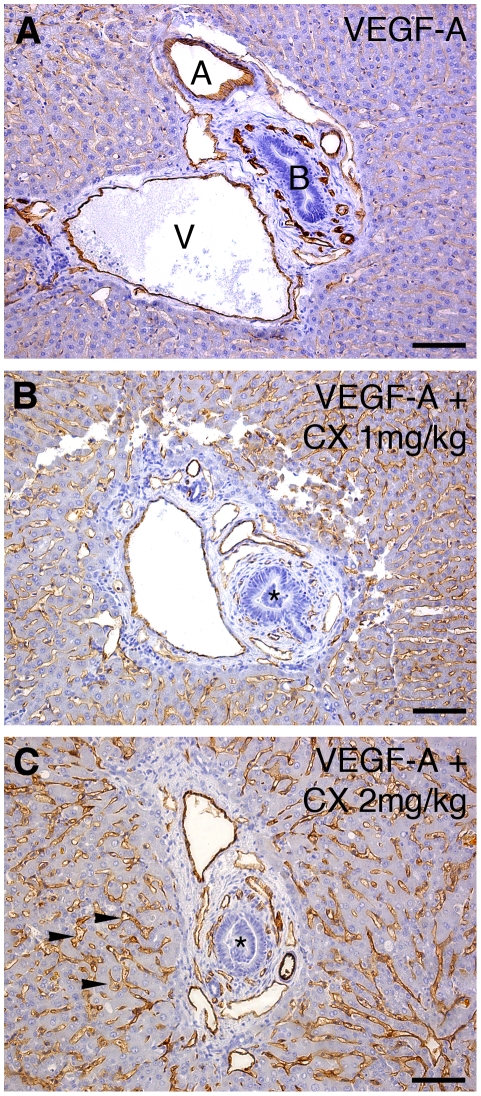
CX treatment alters liver morphology. (A–C) Liver samples from rabbits were stained with CD31/PECAM1 (brown) and counter-stained with hematoxylin (blue). (A) AdVEGF-A + vehicle, (B) AdVEGF-A + 1 mg/kg CX and (C) AdVEGF-A + 2 mg/kg CX. Microphotographs show representative liver portal triads. (B–C) Note the dose dependent dilation of the liver sinusoids (arrowheads in C) as well as the increased staining intensity of CD31 in CX-treated livers. The bile ducts seemed occluded by intraluminal epithelial cells (asterisks in B and C). A: Artery, V: Vein, B: bile duct. Scale bars are 100 µm.

To study the effect of CX on vascular morphogenesis and functionality in a relevant, yet less complex milieu than that of a tumor, we turned to the rabbit hind limb model. In this model, adenoviral (Ad) VEGF-A_165_ gene transfer to the semimembranous muscle led to vessel dilation and proliferation, not seen in skeletal muscle transduced with a control gene, LacZ (**Figure 3**). Contrast pulse sequence (CPS) ultrasound was used to determine the perfusion in living animals [33]. Compared to the control leg, VEGF-A_165_ gene transfer resulted in a more than 10-fold increase of vascular perfusion (**Figure 4**). To determine the dose of CX to be administered, several clinical chemistry markers were analyzed. Even at low doses (1–2 mg/kg), there was a dose-dependent increase in markers for liver stasis and toxicity including serum total bilirubin (s-TB), serum alkaline phosphatase (s-ALP) and serum alanin aminotransferase (s-ALT). In addition, we observed an increase in the levels of serum creatinin, indicating kidney damage (**Table 1**). Histological analysis of the liver, using CD31/PECAM-1 staining, revealed dose-dependent severe dilation of the sinusoids upon CX-treatment, as well as increased staining intensity for CD31. Furthermore, intrahepatic bile ducts were occluded by epithelial structures, a finding in line with the increased s-ALP (**Figure 5**). Consequently, low doses of CX (1–2 mg/kg/day) were chosen for further studies. In comparison to AdVEGF-A-treated skeletal muscle, the combined administration of CX and VEGF-A resulted in dose-dependent blood vessel dilation and most notably an increased number of CD31/PECAM1-positive structures filling the lumen of the vessels (**Table 2**).

**Table 1:** Clinical chemistry parameters of VEGF-A and CX-treated rabbits.

	Day 0			Day 6		
**Gene Transfer**	VEGF-A	VEGF-A	VEGF-A	VEGF-A	VEGF-A	VEGF-A
**Systemic treatment**	vehicle	CX 1 mg/kg	CX 2 mg/kg	vehicle	CX 1 mg/kg	CX 2 mg/kg
**S-ALP (U/L) 71±34**	125±24	105±16	119±20	50±19	197±37**	288±7***
**S-ALT (U/L) 48±13**	37±14	84±22	70±32	17±7	232±67**	282±107**
**S-TB (U/L) <2.5**	<2.5	<2.5	<2.5	<2.5	8±2.6**	30±1.8***
**S-CRP (mg/L) <5**	<5	<5	<5	<5	<5	<5
**S-Creatinin (mM) 95±21**	79±10	79±9.2	88±9.2	89±12	149±26*	155±26*
**S-LD (U/L) 107±44**	73±21	69±11	98±16	127±42	120±19	140±14
**S-Na^+^ (mM) 143±3**	132±6.5	133±3.8	131±13	138±1.0	136±0.81	141±14
**S-K^+^ (mM) 4.2±0.3**	4.6±0.24	3.7±0.19	4.2±0.14	4.3±0.14	4.2±0.35	3.6±0.57

Serum markers of toxicity and inflammation were assayed using blood samples from the rabbit at the beginning (day 0) and end of the study (day 6). Changes in serum levels of the indicated markers were analyzed for statistically significant changes at day 6 between CX and vehicle-treated animals. In CX-treated rabbits there were significant increases of alkaline phosphatase, alanine aminotransferase and total bilirubin indicating liver stasis and toxicity. There was also an increase in creatinin after compound X treatment, suggesting renal toxicity.

Compound X: CX, S: serum, ALP: alkaline phosphatase, ALT: alanine aminotransferase, TB: total bilirubin, CRP: C-reactive protein, LD: Lactate dehydrogenase. Analyte levels are given +/− standard deviatio n. Reference values for rabbit are from Hewitt et al., and Ypsilantis et al.[48], [49]. Significance level using students t-test.

* =  <0.05,

** = P<0.01.

*** = P<0.001.

**Table 2:** Quantification of occluded capillaries in rabbit skeletal muscle upon VEGF-A and CX treatment.

	Number of microscopy fields grouped according to number of occluded capillaries		
	1^st^–33^rd^ percentile (few occluded capillaries)	34^th^–66^th^ percentile	67^th^–100^th^ percentile (many occluded capillaries)
**VEGF-A + vehicle**	17	6	7
**VEGF-A + 1** **mg/kg CX**	11	6	13
**VEGF-A + 2** **mg/kg CX**	2	16	12

The number of occluded capillaries per mm^2^ was determined from photographs of microscopy fields from all treatment groups. These numbers ranged from 3.5 to 88.3 and were used to categorize the microscopy fields into three groups: Microscopy fields within 1^st^–33^rd^ percentile, 34^th^–66^th^ percentile and 67^th^–100^th^ percentile, respectively. The 33^rd^ percentile was at 15.6 and the 67^th^ percentile at 34.6. The number of microscopy fields within each category was then determined and are displayed in the table. VEGF-A+ vehicle versus VEGF-A+ CX (1 mg/kg), Chi-square test P = 0.0002; VEGF-A+ vehicle versus VEGF-A+ CX (2 mg/kg), Chi-square test. P = 0.0033.

Staining for another endothelial marker, podocalyxin, also identified the intraluminal structures as endothelial cells (**Figure 3**). As the vast majority of the vessels follow the fibers of the skeletal muscle, there is little risk of mistaking an occluded vessel from a tangentially sectioned vessel. CX treatment also led to a dose-dependent decrease in vascular perfusion, measured as a reduced CPS signal. Doses of 1 or 2 mg/kg/day of CX reduced CPS-signal by 48% and 66% of control, respectively (**Figure 4**). In addition, blood vessel leakage was determined using the modified Miles assay where extravasated Evans blue (intravenously injected and subsequently bound to plasma proteins) was quantified. Adenoviral gene transfer of VEGF-A led to a 35-fold induction of Evans blue leakage. 2 mg/kg/day of CX treatment resulted in a 76-fold increase in the leakage of the newly formed vessels (**Figure 4**). Importantly, there were no differences in permeability between vehicle treated and CX-treated muscles in the absence of AdVEGF-A treatment.

## Discussion

We noted a pronounced change in vascular architecture after CX treatment in several model systems. During developmental angiogenesis and in the tumor model, the endothelial cell surface was rich in cytoplasmic protrusions and tip cell-like protrusions were frequently observed. In retinal angiogenesis, the hypersprouting led to an increased number of vessels that eventually coalesced into a sheet-like endothelial matt. CX treatment in the RENCA tumor increased the number of blood vessel profiles and the perimeter around the CD31-positive structures. These changes are similar to what is seen after genetic loss or pharmacological inhibition of Dll4 or Notch1. This leads to increased number of endothelial tip cells and hypersprouting both during developmental- and tumor angiogenesis [13], [14], [15], [16], [17], [19], [21], [23], [34]. In addition, we noted loss of ASMA-positive cells around the blood vessels, which previously has been seen after endothelial specific knockout of Jagged-1 or after Dll4 blockade during tumor angiogenesis [22], [35]. We hypothesize that the main effect of CX-mediated inhibition of the gamma-secretase is blockade of Notch signaling which leads to hypersprouting and the formation of an immature vessel network that coalesce into larger vessel structures. Furthermore, an interesting observation in our study was the CD31- or Podocalyxin-positive structures that partially or completely filled out the blood vessel lumen. These structures were very notable in the rabbit hind limb model, where the capillaries are cross-sectioned, but were also evident in the mouse tumor model. Our use of two distinct markers for endothelial cells strongly suggest that the cellular structures filling the vessel lumens are of endothelial origin, but does not rule out contribution from non-endothelial cells. Furthermore, others have reported vessel occlusion following Dll4-inhibition in an *in vitro* model, where HUVEC sprouting in fibrin gels led to the loss of lumen-like structures, which were replaced by densely packed endothelial cells [21]. Similarly it has been shown in the presenilin-1 knockout mice that the brain capillaries are occluded by abnormally shaped endothelial cells, which occasionally form multilayered stacks of endothelial cells completely filling the vessel lumen [36]. We speculate that the loss of Notch signaling leads to hyperproliferation of endothelial cells, which fail to organize properly and thereby occlude the newly formed blood vessels. Moreover, we observed that vascular leakage increased after GSI treatment. This could be explained by the known inhibitory effect of Notch on VEGF-A activity, since VEGF-A is known to potently induce vascular leakage, reviewed in [37]. Furthermore, CX had no effect on vascular leakage on its own further arguing that inhibition of Notch signaling rather potentiate the effects of VEGF-A. Alternatively, the abnormal vascular structures triggered by GSI treatment could lead to reduced vascular integrity and increased vascular leakage. Taken together, the abnormal vessel architecture in combination with increased vascular leakage, could explain the loss of vascular perfusion after CX treatment. Notably, we administered 10 mg/kg/day to the mice without overt toxicity, and the doses used in rabbits (1–2 mg/kg) were thus likely in the low range in terms of modulating vessel growth. Therefore, had we not observed symptoms of toxicity in rabbit model, higher dosing would have been possible, with potentially even more dramatic changes in vascular morphology.

In the tumor model, it is interesting to note that the occluded vessels were most commonly observed in proximity of necrotic areas. This could suggest that vessel occlusion led to loss of blood vessel perfusion, which deprived the tumor of oxygen and nutrients. However, we were not able to quantify any differences in proliferation, apoptosis, or necrosis between CX and vehicle treated tumors at the end of the study (day 21). Small additive differences in the measured parameters could still lead to the end result i.e. smaller tumors in the GSI treated group. Another plausible explanation is a progressive tumor adaptation to the treatment, in turn leading to immeasurable differences at day 21.

Early work by Paris and co-workers indicated that GSIs might have an anti-angiogenic effect on endothelial cells *in vitro* and *in vivo*, and inhibited tumor growth [38]. However, in contrast to that study, but in line with the recent genetic and pharmacological studies mentioned above, we found that CX promoted angiogenic sprouting and vessel growth *in vitro* and during developmental angiogenesis. The contrasting findings between our study and the one by Paris and co-workers could be explained by the use of different tumor models or different GSIs - where one of them does not even inhibit Notch processing (JLK6) [39]. There were also significant differences in administration, dosing of the GSIs, and finally the fact that Paris et al. analyzed smaller sized tumors than in the present study.

GSI treatment is unfortunately associated with side effects. One of the better-studied side effects is increased mucus production leading to diarrhea, which stems from increased proliferation of mucus producing Goblet cells, due to Notch inhibition [31]. In addition, blockade of Notch results in altered lymphocyte development including decreased number of mature B-cells [40]. Recently, it has also been shown that Dll4 or combined Notch1 and -2-inhibition can lead to development of vascular tumors and hepatotoxicity [23], [41]. Similarly, we found that CX treatment led to severe hepatotoxicity in rabbits. This effect is likely triggered in part by abnormal activation of liver endothelial cells as suggested by Yan et al and Wu et al [23], [41]. In addition, we observed abnormal bile ducts and detected increased ALP-levels, indicative of cholestasis. Interestingly, Notch signaling is important not only for endothelial cells, but also for bile duct epithelial cells. For example, Notch2, but not Notch1, has been shown to be indispensable for normal perinatal and postnatal intrahepatic bile duct development [42]. In addition, we found signs of renal toxicity, which could be a GSI-class effect, alternatively toxicity specific for CX. Despite these side effects, GSIs is still an interesting class of drugs when compared to more selective agents as therapeutic antibodies. GSIs have been shown to affect not only tumor cell growth via Notch, but also other targets e.g. erbB-4, mToR and Glut1 and thereby affecting other signaling pathways important for tumor growth, nutritional status of the tumor and possibly cancer stem cells [27], [28]. Importantly, the gastrointestinal side effects mediated by increased Goblet cell number have been shown to be manageable using altered dosing regiments or concomitant glucocorticoid treatment [30], [43].

In conclusion we have shown that the GSI Compound X, promotes angiogenic sprouting and neovascular formation during development and tumor growth. Using a model of VEGF-A-driven angiogenesis in the rabbit hind limb, we showed that CX treatment resulted in vessel occlusion, reduced perfusion and increased vascular leakage. We hypothesize that GSI treatment leads to endothelial hyperproliferation and subsequent occlusion of newly formed blood vessels, which leads to reduced vascular perfusion. This vascular hypersprouting, in combination with increased vascular leakage, then leads to oxygen and nutrient deprivation of the tumor.

VEGF-A driven angiogenesis is important for tumor oxygen and nutrient supply. We have shown in models of VEGF-A-driven angiogenesis that a GSI, inhibiting the Notch pathway, can strongly affect vascular density, perfusion, and leakage. These observations warrant further investigations of the role GSIs might have in modulating tumor angiogenesis. Several compounds targeting Notch signaling are under development for treatment of a diverse array of malignancies [19], [23], [29], [30], [34], [44]. Each of the compounds has its pros and cons in terms of selectivity and efficacy in blocking the Notch pathway. Further research will determine which one of these compounds that ultimately can be used effectively in the clinic.

## Materials and Methods

### Ethics statement

The developmental angiogenesis study was performed under the license number: 354–04, granted by the Animal Ethics Committee, Göteborg, Sweden.

The tumor study was performed using 8–10 weeks old female BALB/c mice. Experimental protocols had been approved by the Ethics Committee for Animal Experimentation according to the United Kingdom Coordinating Committee on Cancer Research Guidelines. The experimental protocol was registered by the Regierungspräsidium Freiburg (G-04/50).

The rabbit study was performed under the license number: ISLH-2004-03492/Ym-23, study approved by University of Kuopio Ethical Committee and license granted by County Administrative Board.

### Spheroid-based cellular angiogenesis assay

The Gamma-secretase inhibitor CX was synthesized as described in [31]. The Spherogenex assay was performed as described in [32]. Briefly, HUVECs were purchased from PromoCell, Heidelberg, Germany, and cultured according to the manufacture's instructions. HUVECs clustered into spheroids in cell culture media over night and dispensed in a 3-dimensional collagen type I matrix. Spheroids were assayed for sprouting in the presence of different concentration of CX for 24h and the ten longest sprouts from 10 spheroids were summarized as cumulative sprout length (CSL).

### Immunohistochemistry of retinal vasculature

CX was dissolved in vehicle (10% ethanol and 90% corn oil) and administered subcutaneously 5–20 mg/kg/day postnatal day 3 and 4. The eyes were isolated and retinas dissected on day 5. Paraformaldehyde fixation and staining using isolectin B4 was done as previously described [13].

### RENCA tumor model

The study consisted of 2 groups containing 12 female BALB/c mice each. At day 0, RENCA cells were orthotopically implanted into the left kidney of all mice. Starting the following day (day 1) animals in the treatment group received 10 mg/kg CX per os (p.o.) once a day for the duration of 21 days. Animals of the control group received vehicle (10% Ethanol, 90% corn oil) also once a day p.o. starting day 1 until day 21.

At day 21 the study was terminated and a necropsy performed. At necropsy, primary tumor weight and volume was determined.

For histological examination of the tumor vasculature, cryosections of primary tumor tissues (thickness  =  5–10 µm) were taken from all animals. For the visualization of the blood vessels, immunohistochemical staining for CD 31 (1∶500 PECAM-1 Cat. 553370, PharMingen, San Diego, CA) was performed, and vessels were counted microscopically using a defined magnification (x200), control treated (n = 11) and CX-treated (n = 9). For fluorescent images, Alexa Fluor 488 donkey anti-rat IgG, (1∶200, A21208, Molecular Probes) was used. To stain for podocalyxin, goat anti-podocalyxin (1∶100, AF1556, R&D Systems, Minneapolis, MN) and donkey anti-goat IgG Alexa 488 (1∶200, A-11055, Molecular Probes) were used. Monoclonal Anti-Actin, α-Smooth Muscle – Cy3 antibody, (1∶100, C6198 Sigma, Saint Louis, MO, USA) was used to visualize mural cells.

In addition, the proliferation index of the tumor tissue was examined by BrdU labeling of the cryosections. For this purpose, BrdU (500 mg/kg) was administered 12 h before sacrificing the animals. Apoptotic index was determined using TUNEL (Cat. 12 156 792 910, Roche, Mannheim, Germany) and cleaved Caspase 3 stainings of tumor sections. Cleaved caspase-3 (1∶500 #9661, Cell Signaling Techn, Danvers, MA) and Alexa Fluor 555 donkey anti-rabbit IgG (1∶200 # A-31572, Molecular Probes, Eugene, OR) were used. The determination of the necrotic areas within the primary tumor was performed using the CD 31-stained tissue samples. Necrotic areas were identified as areas in which no vessels could be detected.

### Rabbit hind-limb model

#### Gene transfer and GSI administration

New Zealand White (NZW) rabbits (3.0–3.5 kg) were used for gene transfers. Intramuscular gene transfers with adenoviral human VEGF-A (n = 9) and AdLacZ (n = 1) were done into the hindlimb semimembranosus muscle with method as previously described [45], [46]. Rabbits were anesthetized with medetomidine (Domitor 0,3 mg/kg, Orion, Finland) and ketamine (Ketalar 20 mg/kg, Pfizer, Finland) and the total dose of 1×10^11^ virus particles in 1 ml was divided to ten 100 µl injections. CX (1 mg/kg (n = 3) or 2 mg/kg (n = 3)) or vehicle (n = 3) was administered by subcutaneous injections once a day. Animals were euthanized six days after gene transfer.

#### Ultrasound imaging

Skeletal muscle perfusion was measured with Acuson Sequoia and Cadense Pulse Sequence (Siemens) ultrasound imaging using 15L8 transducer of gene transferred semimembranosus muscle and the contralateral intact muscle, respectively[33]. Images were taken from transversal sections after a single bolus (0.5 ml) of SonoVue contrast agent (Bracco, The Netherlands). Measurements were performed six days after gene transfer. Perfusion ratio between gene transduced muscle and intact muscle was quantified with DataPro 2.13 software (Noesis) using the maximum signal intensities.

#### Modified Miles Assay

Modified Miles assay was used to evaluate tissue edema [47]. Evans Blue dye (30 mg/kg) was injected 30 min before euthanasia via ear vein. Animals were perfusion-fixed with 1% paraformaldehyde in 0.05 M citrate buffer (pH 3.5) via left ventricle after euthanasia. Muscles were photographed, and samples were taken and weighted from both gene transferred muscle and contralateral intact muscle. Evans Blue dye was eluted in formamide and measured with spectrophotometer of absorbance at 610 nm. Absorbance was normalized to the sample weight and ratio between gene transferred and intact muscle samples was calculated.

#### Histology of rabbit skeletal muscle

7 µm thick paraffin sections were stained with monoclonal antibody against CD31 (1∶50, clone JC/70A, Dako, Glostrup, Denmark) and against podocalyxin-like 1 (1∶200, sc-23903, Santa Cruz Biotechnology, Santa Cruz, CA). Signal detection was done with avidin-biotin-horse radish peroxidase (HRP) system with diaminobenzidine (DAB) (Vector Laboratories/LabVision). Microphotographs were taken with an Olympus AX70 microscope (Olympus Optical, Tokyo Japan) and with a ZEISS AxioImager.M2 (Zeiss, Jena, Germany).

#### Quantification of occluded capillaries

A capillary was considered occluded, and thus counted, if more than 50% of the luminal area was filled with CD31 positive cells. Ten microscopy fields (200×magnification) per animal were captured. Altogether 30 microscopy fields from each treatment group were used for the quantification. Occluded capillaries were counted from the proximity of the needle track in each muscle biopsy.

### Statistical analysis

Student's t-test was performed for comparison of two groups and ANOVA was performed for analysis of three groups. The t-test was calculated as unpaired and two-tailed, except for the analysis of ASMA-coated vessels that was calculated as one-sided. This was based on the assumption that there would be a reduction in the ASMA-positive cells in the treatment groups based on the publication by Scehnet et al.[22]. Alpha values less than of 0.05 were regarded as significant. The Chi-square test was used when analyzing the frequency of occluded vessels. The GraphPad Prism software v. 5.01 was used for all statistical analysis.

## Supporting Information

Figure S1 High resolution confocal images of vessels in the CX treated tumors. RENCA tumors stained for Podocalyxin or CD31 (green), and DAPI (blue or white). (A) A vehicle treated vessel as reference to B–D. (B) An example of a CX-treated tumor vessel filled with podocalyxin-positive cells with blue DAPI-stained nucleus, white asterisks. (C) Same image as B, where the green channel was removed to more clearly see the nuclei, black asterisks (DAPI in white). (D) A CX-treated tumor showing a partially occluded lumen, filled with CD31-positive cells. Scale bars 25 µm.
(TIF)
