# Supplementary figures and images for: Gamma-Secretase Inhibitor Treatment Promotes VEGF-A-Driven Blood Vessel Growth and Vascular Leakage but Disrupts Neovascular Perfusion
Source: PLoS One. 2011 Apr 14;6(4):e18709. doi: 10.1371/journal.pone.0018709 (PMC3077402; doi:10.1371/journal.pone.0018709)

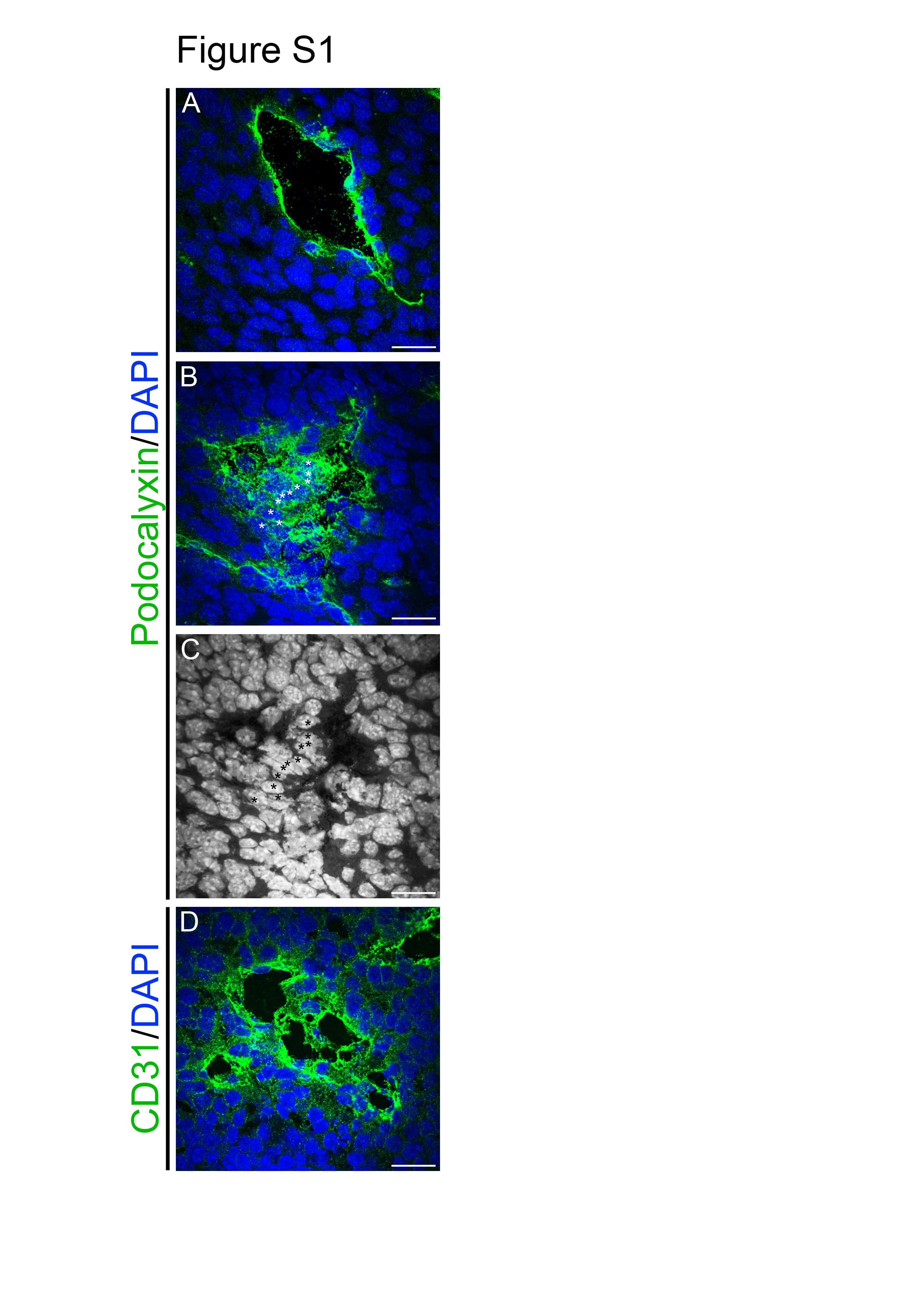

Supplement: Figure S1 — High resolution confocal images of vessels in the CX treated tumors. RENCA tumors stained for Podocalyxin or CD31 (green), and DAPI (blue or white). (A) A vehicle treated vessel as reference to B–D. (B) An example of a CX-treated tumor vessel filled with podocalyxin-positive cells with blue DAPI-stained nucleus, white asterisks. (C) Same image as B, where the green channel was removed to more clearly see the nuclei, black asterisks (DAPI in white). (D) A CX-treated tumor showing a partially occluded lumen, filled with CD31-positive cells. Scale bars 25 µm. (TIF) [file pone.0018709.s001.tif]
